# Attenuation of Cigarette-Smoke-Induced Oxidative Stress, Senescence, and Inflammation by Berberine-Loaded Liquid Crystalline Nanoparticles: In Vitro Study in 16HBE and RAW264.7 Cells

**DOI:** 10.3390/antiox11050873

**Published:** 2022-04-28

**Authors:** Keshav Raj Paudel, Nisha Panth, Bikash Manandhar, Sachin Kumar Singh, Gaurav Gupta, Peter R. Wich, Srinivas Nammi, Ronan MacLoughlin, Jon Adams, Majid Ebrahimi Warkiani, Dinesh Kumar Chellappan, Brian G. Oliver, Philip M. Hansbro, Kamal Dua

**Affiliations:** 1 Centre for Inflammation, Centenary Institute and University of Technology Sydney, Faculty of Science, School of Life Sciences, Sydney, NSW 2007, Australia; keshavraj.paudel@uts.edu.au (K.R.P.); n.panth@centenary.org.au (N.P.); 2 Discipline of Pharmacy, Graduate School of Health, University of Technology Sydney, Sydney, NSW 2007, Australia; bikash.manandhar@uts.edu.au; 3 Faculty of Health, Australian Research Centre in Complementary and Integrative Medicine, University of Technology Sydney, Ultimo, NSW 2007, Australia; sachin.16030@lpu.co.in (S.K.S.); jon.adams@uts.edu.au (J.A.); 4 School of Pharmaceutical Sciences, Lovely Professional University, Jalandhar-Delhi GT Road, Phagwara 144411, Punjab, India; 5 School of Pharmacy, Suresh Gyan Vihar University, Jaipur 302017, Rajasthan, India; gaurav.gupta@mygyanvihar.com; 6 Uttaranchal Institute of Pharmaceutical Sciences, Uttaranchal University, Dehradun 248007, Uttarakhand, India; 7 School of Chemical Engineering, University of New South Wales, Sydney, NSW 2052, Australia; p.wich@unsw.edu.au; 8 Australian Centre for NanoMedicine and Centre for Advanced Macromolecular Design, University of New South Wales, Sydney, NSW 2052, Australia; 9 School of Science, Western Sydney University, Penrith, NSW 2751, Australia; s.nammi@westernsydney.edu.au; 10 IDA Business Park, H91 HE94 Galway, Connacht, Ireland; rmacloughlin@aerogen.com; 11 School of Pharmacy & Biomolecular Sciences, Royal College of Surgeons in Ireland, D02 YN77 Dublin, Leinster, Ireland; 12 School of Pharmacy & Pharmaceutical Sciences, Trinity College, D02 PN40 Dublin, Leinster, Ireland; 13 School of Biomedical Engineering, University of Technology Sydney, Sydney, NSW 2007, Australia; majid.warkiani@uts.edu.au; 14 Institute for Biomedical Materials and Devices, Faculty of Science, University of Technology Sydney, Sydney, NSW 2007, Australia; 15 Department of Life Sciences, School of Pharmacy, International Medical University, Kuala Lumpur 57000, SGR, Malaysia; dinesh_kumar@imu.edu.my; 16 Woolcock Institute of Medical Research, University of Sydney, Sydney, NSW 2037, Australia; brian.oliver@uts.edu.au; 17 University of Technology Sydney, Faculty of Science, School of Life Sciences, Sydney, NSW 2007, Australia

**Keywords:** cigarette smoking, airway inflammation, oxidative stress, senescence, berberine, liquid crystalline nanoparticles

## Abstract

Cigarette smoke is considered a primary risk factor for chronic obstructive pulmonary disease. Numerous toxicants present in cigarette smoke are known to induce oxidative stress and airway inflammation that further exacerbate disease progression. Generally, the broncho-epithelial cells and alveolar macrophages exposed to cigarette smoke release massive amounts of oxidative stress and inflammation mediators. Chronic exposure of cigarette smoke leads to premature senescence of airway epithelial cells. This impairs cellular function and ultimately leads to the progression of chronic lung diseases. Therefore, an ideal therapeutic candidate should prevent disease progression by controlling oxidative stress, inflammation, and senescence during the initial stage of damage. In our study, we explored if berberine (an alkaloid)-loaded liquid crystalline nanoparticles (berberine-LCNs)-based treatment to human broncho-epithelial cells and macrophage inhibits oxidative stress, inflammation, and senescence induced by cigarette-smoke extract. The developed berberine-LCNs were found to have favourable physiochemical parameters, such as high entrapment efficiency and sustained in vitro release. The cellular-assay observations revealed that berberine-LCNs showed potent antioxidant activity by suppressing the generation of reactive oxygen species in both broncho-epithelial cells (16HBE) and macrophages (RAW264.7), and modulating the genes involved in inflammation and oxidative stress. Similarly, in 16HBE cells, berberine-LCNs inhibited the cigarette smoke-induced senescence as revealed by X-gal staining, gene expression of CDKN1A (p21), and immunofluorescent staining of p21. Further in-depth mechanistic investigations into antioxidative, anti-inflammatory, and antisenescence research will diversify the current findings of berberine as a promising therapeutic approach for inflammatory lung diseases caused by cigarette smoking.

## 1. Introduction

Chronic obstructive pulmonary disease (COPD) is a disease that affects the lungs. The disease is characterised by chronic airway inflammation and impairment of lung function due to damage in the lung architecture leading to airflow limitation [1]. Cigarette smoking is the main risk factor of COPD, and chronic smoking is associated with airway inflammation, change in respiratory bacterial microbiome, and fibrosis [2,3]. Cigarette smoke contains thousands of compounds that are carcinogenic, oxidative, and inflammatory in nature [4]. Clinical studies have proven that various body fluids such as urine and serum, lung tissue, and exhaled breath of cigarette smokers have significantly higher levels of oxidants such as 8-iso-prostaglandin F_2-alpha_ (F2 isoprostane), 3-nitrotyrosine, 4-hydroxy-2,3-nonenal, malondialdehyde, 8-oxo-7,8-dihydro-20-deoxyguanosine, 8-oxo-7,8-dihydro-20-deoxyadenosine, and thymine glycol and 5-hydroxyuracil [5,6]. The expression of several proteins is impaired in COPD patients. For instance, the increased level of urokinase-type plasminogen activator receptor (uPAR) in the sputum of COPD patients is associated with airway limitation [7]. Another study reported higher epidermal growth factor (EGF) expression in damaged epithelium (1.4–1.8 times; *p* ≤ 0.05) of ex-smokers with COPD compared with ex-smokers without COPD [8]. In comparison to healthy subjects, the level of circulating growth-differentiation protein 15 (GDF-15) is 2.1-fold higher in COPD patients [9]. Osteopontin (OPN) is a protein detected on the surface of small airway epithelial cells, and it plays a crucial role in the development of inflammation via recruitment of neutrophils and tissue remodelling. Studies have shown that the levels of OPN in tissue of moderate-to-severe COPD is higher compared to healthy controls. In vitro studies also suggest that OPN expression is upregulated in submerged basal cell cultures exposed to cigarette-smoke extract (CSE) [10]. Therefore, drugs targeting these proteins (uPAR, EGF, GDF-15, OPN) could be a promising approach in the management of COPD.

During the progression of COPD, dysregulation of various enzymes such as myeloperoxidase, NADPH oxidase, lipoxygenases, xanthine oxidase, nitric oxide synthase, and cytochromes P450 involved in the generation of oxidative stress, and antioxidant enzymes such as superoxide dismutase, catalases, and glutathione peroxidase involved in protection from oxidants result in oxidative damage of the lung architecture. This is mediated by various processes such as lipid peroxidation, protein oxidation, DNA and RNA damage, mitochondrial damage, and ferroptosis [6]. This clinical feature is also correlated with in vitro study where exposure of human broncho-epithelial cell line (16HBE) to 2% CSE had resulted in the significant production of reactive oxygen species (ROS). This further leads to activating the PI3K-AKT-mTOR signalling pathway and senescence of 16HBE by upregulating p16 and p21 protein expression [11]. Cigarette smoke is also reported to induce inflammatory mediators such as nitric oxide; tumor necrosis factor-alpha (TNF-α); IL (interleukin)-6, IL-1β, IL-8, and GM-CSF from alveolar macrophage [12,13]; and CXCL-8, IL-1β, IL-6 from human broncho-epithelial cells [14]. As in various clinical studies, in vivo animal model studies (mice exposed to cigarette smoke) and in vitro model studies (cell lines exposed to CSE) have already shown that tobacco smoke is toxic to human, animal, and cell lines as they induce oxidative stress, apoptosis, senescence, and massive release of inflammatory cytokines/chemokines; therefore, a drug candidate with potent antioxidative and anti-inflammatory activity that can slow down premature cellular senescence and inflammation is desirable to halt the progression of chronic lung diseases such as COPD. To date, several anti-inflammatory drugs including corticosteroids are prescribed to patients diagnosed with chronic airway inflammation (bronchitis or COPD). However, the benefit-to-risk ratio of these drugs is not favourable to the patient due to several issues such as side effects, cost, and the complexity of using a specific medication (example: inhaler). In this context, a nutraceutical-based approach for the mitigation of airway inflammation in respiratory diseases is gaining considerable attention due to its potency, fewer side effects, ease of use, and affordability [15].

Plants such as *Eriobotrya japonica* (loquat) [16], *Nelumbo nucifera* (lotus) [17], *Camellia sinensis* (green tea) [18], *Punica granatum* (pomegranate) [19], and their extracts possess antioxidant and anti-inflammatory activities, and therefore are considered beneficial in the management of inflammatory lung diseases such as asthma, CODP, pulmonary fibrosis, and lung cancer. Apart from plant extracts, their isolated single biocompounds such as rutin, naringenin, boswellic acid, and berberine have shown potent activity in various lung disease models [20,21,22,23,24]. Berberine is an iso-quinoline alkaloid, primarily found in the plant families of Ranunculaceae and Papaveraceae [25]. Considerable scientific investigations have justified the anti-inflammatory potential of berberine in various cell lines [26]. Synthetic derivatives of berberine, for example, dimethyl berberine, inhibit reactive oxygen/nitrogen species, mitochondrial dysfunction, and inflammatory mediators such as NFκB, TNF-α, IL-6, and IL-8 [27]. Although berberine exerts numerous beneficial activities against a range of ailments, its application as a therapeutic agent is limited due to several bottlenecks and stumbling issues such as poor oral bioavailability, low gastrointestinal absorption, and a high degree of elimination [28,29]. To achieve the ideal therapeutic outcome, it is essential to improve its physicochemical properties, such as solubility, bioavailability, and maintaining a desired plasma therapeutic concentration.

Utilising the advances in nanotechnology, various nanoformulation-based drug-delivery systems are now being employed and some are undergoing research as a potential strategy for lung diseases [30]. Recent developments in nanotechnology-based therapeutic approaches have created a positive hope to rediscover novel therapeutic strategies for the management of various lung diseases [31]. Among nanoformulations, liquid crystalline nanoparticles (LCNs) are a type of drug carrier with considerable interests among researchers and pharmaceutical sectors due to their versatility in improving bioavailability and enhancing the stability of therapeutic compounds [32]. In addition, these nanostructures have the inherent ability to alter the release of drugs when administered through various routes [20]. We previously reported that berberine-loaded LCNs (BBR-LCNs) exhibit potent anticancer activity in human lung epithelial carcinoma (A549) cell lines by inhibiting proliferation and migration [33,34]. Using the same formulation at a safe dose for healthy human bronchial epithelial cell lines (16HBE) and macrophage cell lines (RAW264.7), we investigated the protective effects of BBR-LCN formulation against cigarette-smoke-induced oxidative stress, inflammation, and senescence in this study.

## 2. Materials and Methods

### 2.1. Formulation and Physiochemical Characterisation of BBR-LCNs

The formulation of BBR-LCNs and its physiochemical characterisation, such as particle size, polydispersity index, zeta potential, entrapment efficiency, morphology, and in vitro release study, were carried out and the data were published in our recent publication [33].

### 2.2. Cell Culture and Reagents

Cell-culture experiments (in vitro) were carried out using the healthy human broncho-epithelial cell line; 16HBE (American Type Culture Collection (Manassas, VA, USA), which was a kind gift by Prof. Qihan Dong at Charles Perkin Centre, The University of Sydney, Sydney, Australia, and RAW264.7 cells (macrophage cell line) were purchased from ATCC, USA. The cells were cultured in a standard 5% CO_2_ incubator in a Dulbecco’s Modified Eagle’s Medium plus 5–10% fetal bovine serum and 1% antibiotic mix (penicillin and streptomycin). Cells were regularly tested for mycoplasma contamination and all experiments were carried out using mycoplasma-negative cells. MTT (3-[4,5-dimethylthiazol-2-yl]-2,5-diphenyl tetrazolium bromide), dimethyl sulphoxide (DMSO), dichlorodihydrofluorescein diacetate (DCF-DA), were procured from Sigma-Aldrich, St. Louis, MO, USA. The Griess reagent kit, for nitrite quantification (G7921), was purchased from ThermoFisher, Australia. Anti-p21 antibody (2947S) was purchased from Cell Signalling Technology, Victoria, Australia. Beta galactosidase staining (X-gal) kit (ab102534) and goat antirabbit Alexa647 (ab150079) antibody were purchased from Abcam, Victoria, Australia.

### 2.3. Preparation of Cigarette-Smoke Extract (CSE)

One research-grade reference cigarette 3R4F from Kentucky University, USA was burned, and the resultant smoke was bubbled through 10 mL PBS. This was considered as 100% CSE. The 100% CSE was passed through the 0.22 μm filter and further diluted to 5% with cell-culture media. For uniformity, we measured the absorbance of freshly prepared 100% CSE each time, and CSE with similar absorbance value was used for various in vitro assay. The freshly prepared CSE was used on the exposed cells within 30 min. Different batches of reference cigarettes (for research purpose only) prepared by Kentucky University, such as 2R4F and 3R4F, are widely used by researchers across the globe. Literature suggests that exposing 16HBE and RAW264.7 cells with 5% CSE (some use 2–10%) induced inflammation, oxidative stress, and senescence [13,35,36].

### 2.4. MTT Assay (Cell-Viability Assay)

The MTT colorimetric assay was utilised to study the toxicity of CSE and BBR-LCNs on both 16HBE and RAW264.7 cells, as previously described by Lee et al. (2016) [37]. Both cell lines were seeded at 10,000 cells per well in a 96-well plate. After overnight attachment, cells were pretreated for 1 h with/without various doses of BBR-LCNs (0.1, 1, 2.5, 5, and 10 μM) followed by exposure to 5% CSE for next 24 h. Afterwards, MTT solution (5 mg/mL stock) was added at 10 µL to each well and incubated for next 4 h. The culture media were removed and the formazan crystals (developed by enzymatic activity of live cells on MTT) were dissolved in 100 μL dimethyl sulfoxide. The absorbance of the purple-coloured product was quantified at 540 nm using a microplate reader (POLARstar Omega, purchased through BMG LABTECH Pty. Ltd., Victoria, Australia). The viability of the control cells (only media-treated, no CSE and BBR-LCNs) was normalised to 100%, and the percentage viability of BBR-LCNs and 5% CSE-treated cells was calculated.

### 2.5. Total Cellular Reactive Oxygen Species Assay

#### 2.5.1. Fluorescence Intensity Quantification

16HBE and RAW264.7 cells were plated separately in a black 96-well plate (Greiner CELLSTAR^®^, M0312, Greiner Bio-One GmbH, Frickenhausen, Germany). BBR-LCNs were pretreated with the cells at indicated doses for 1 h followed by exposure to 5% CSE for another 24 h. Then, 10 μM of DCF-DA was added to each well and incubated for 30 min under dark conditions. The fluorescence intensity was quantified at the manufacturer’s recommended excitation wavelength of 488 nm and emission wavelength of 525 nm using a FLUOstar Omega (BMG LABTECH Pty Ltd., Victoria, Australia) [24].

#### 2.5.2. Fluorescence Imaging

16HBE and RAW264.7 cells were cultured in a cover slip inside a 6-well plate. After overnight attachment, the cells were pretreated with BBR-LCNs at different doses for 1 h followed by exposure to 5% CSE for 24 h. Cells were then washed twice with PBS and incubated with 10 μM of DCF-DA for 30 min in dark condition. After washing the cells twice with PBS, microscopic images at 20× magnification (for 16HBE) and 40× (for RAW264.7) were captured immediately using a fluorescence microscope (Zeiss Axio Imager Z2, Oberkochen, Germany) [24].

### 2.6. Senescence Assay

#### 2.6.1. X-Gal Staining

16HBE cells were grown in a glass cover slip inside 6-well plates. Cells were pre-treated with BBR-LCNs at 5μM concentration for 1 h followed by exposure to 5% CSE for another 24 h. Cells were then washed with PBS and fixed with fixative solution (supplied in the kit, ab102534) for 10 min at room temperature. After washing with PBS, the cells were then stained with the staining mixture (staining solution, staining supplement, and X-gal) overnight at 37 °C inside the incubator. Cover slips were transferred from 6-well plates to glass slides and images of cells were captured with Zeiss Axio Imager Z2 microscope at 20× magnification [11].

#### 2.6.2. Immunocytochemistry of p21

16HBE cells were cultured on a cover slip inside a 6-well plate. Cells were pretreated with BBR-LCNs at 5 μM concentration for 1 h followed by exposure to 5% CSE for another 24 h. After washing cells with PBS, fixing with 4% paraformaldehyde for 10 min, permeabilised with 0.5% Triton X-100 for 30 min, blocking with 1% bovine serum albumin for another 30 min, the cells were incubated with anti-p21 (Cell Signalling Technology, 2947S) at 1:800 dilution overnight at 4 °C and next day with goat antirabbit Alexa488 (Abcam, ab150077, Victoria, Australia) at 1:1000 dilution for 1 h. Cover slips were mounted with fluoro mount containing 4′,6-Diamidino-2-phenylindole (DAPI) for nuclear stain, images of cells were taken with Zeiss Axio Imager Z2 microscope (Oberkochen, Germany) at 40× magnification, and mean fluorescence intensity was quantified using Image J software [11].

### 2.7. Human Cytokines Protein Array

The protein array of cytokines 16HBE cells treated with/without 5% CSE and BBR-LCNs was carried out as described in our previous published study [33]. The total protein from 16HBE was lysed with RIPA lysis buffer and extracted for quantified by a Pierce^TM^ BCA protein assay kit (catalogue 23225). Equal amount of protein was loaded for each group to develop the blots using R&D Systems Proteome Profiler Human XL Cytokine Array Kit (R&D Systems, Minneapolis, MN, USA) according to the manufacturer’s instructions.

### 2.8. Nitric Oxide (NO) Assay

Quantification of NO (in terms of nitrite) production from RAW264.7 was carried out using standard Griess reagent method [38]. RAW264.7 cells seeded in 96-well plates were pretreated with various concentrations of BBR-LCNs and exposed to 5% CSE for another 24 h. The culture supernatant media was mixed with Griess’ reagent at 1:1 ratio (100 μL each). The optical density of the colour product was measured by taking absorbance at 540 nm using FLUOstar Omega Reader (BMG LABTECH Pty. Ltd., Victoria, Australia). The level of the nitrite in the supernatant was quantified with respect to the reference absorbance value obtained after serial dilution of NaNO_3_.

### 2.9. Real Time-qPCR (Oxidative Stress, Senescence, and Inflammation Gene)

16HBE and RAW264.7 cells were grown in six-well plates. Cells were pretreated for 1 h with/without various concentrations of BBR-LCNs followed by exposure to 5% CSE for another 24 h. The total RNA was then isolated with a Trizol method. Then, cDNA was synthesised by reverse transcription of RNA (200 ng) followed by real-time quantitative PCR analysis which was carried out for the measurement of gene expression (Table 1). The gene expressions were calculated with the help of 2^−[ΔΔ]Ct^, corresponding to the respective reference genes (GAPDH for 16HBE and HPRT for RAW264.7). Findings were presented as relative abundance with respect to control cells (5% CSE and BBR untreated) [21].

### 2.10. Statistical Analysis

Data are presented as mean ± SEM. Statistical analyses were performed by one-way ANOVA followed by Dunnett’s or Tukey’s multiple comparison test using the Graph Pad Prism software (version 9.3). Statistical significance was accepted at *p* < 0.05.

## 3. Results

### 3.1. Preparation and Physicochemical Characterisation of BBR-LCNs Formulation

The preparation and physicochemical characterisation details of BBR-LCNs formulation were published in our recent publication [33].

### 3.2. Viability of BBR-LCNs Treated 16HBE and RAW264.7 Cells

**Figure 1:**
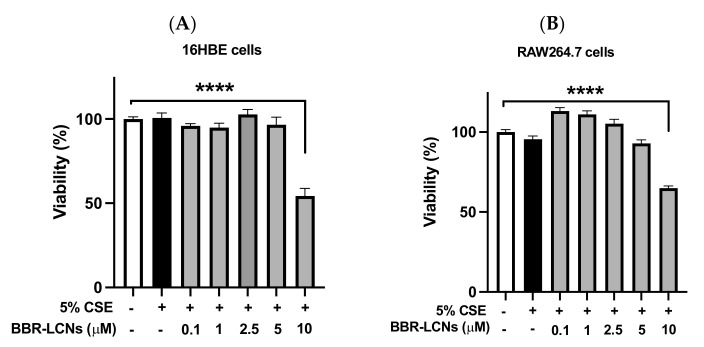
Effect of BBR-LCNs in 16HBE and RAW264.7 cell viability. Cell viability was quantified by MTT colorimetric assay by measuring the absorbance of purple formazan at 540 nm. (**A**) 16HBE cells, (**B**) RAW264.7 cells. **** *p* < 0.0001 vs control (without BBR-LCNs and 5% CSE treatment). Values are expressed as mean ± SEM, *n* = 3 independent experiments. Analysis was performed with one-way ANOVA followed by Dunnett’s multiple comparison test. CSE: Cigarette smoke extract; BBR-LCN: Berberine-liquid crystalline nanoparticles.

The toxicity studies of various doses of BBR-LCNs on the 16HBE cell and RAW264,7 along with their viabilities are shown in Figure 1A,B, respectively. BBR-LCNs, at doses of 0.1–5 μM were safe to both 16HBE and RAW264.7 while the dose of 10 μM was observed to be toxic with a significant decrease in cell viability. Therefore, all in vitro assays were performed in both cell lines with a dose of BBR-LCNs not exceeding 5 μM.

### 3.3. Inhibition of CSE Induced ROS Generation in 16HBE Cells by BBR-LCNs

**Figure 2:**
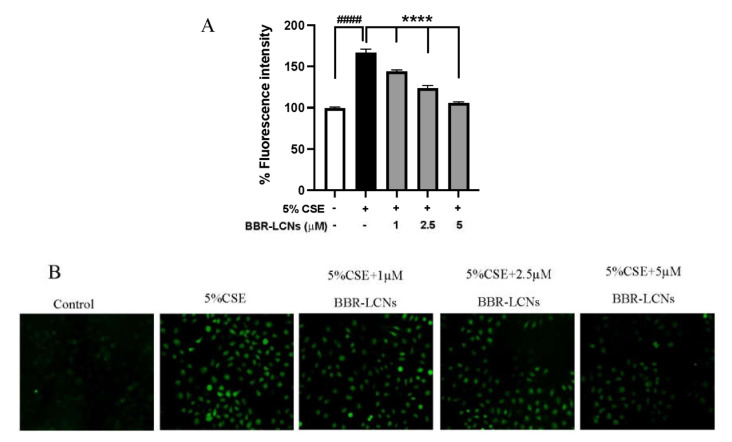
Effect of BBR-LCNs on ROS production in 16HBE cells. (**A**) Measurement of fluorescence intensity. The fluorescence intensity was measured at an excitation 485 nm and an emission 535 nm; n = 3 independent experiments and each independent experiment contained 6 replicates. ^####^ *p* < 0.0001 vs. control (without BBR-LCNs and 5% CSE treatment) and **** *p* < 0.0001 vs. 5% CSE. Values are expressed as mean ± SEM. Analysis was carried out using one-way ANOVA followed by Dunnett’s multiple comparison test. (**B**) Fluorescence imaging. The DCF-DA fluorescence staining images of 16HBE cells treated with 5% CSE and with/without different concentrations of BBR-LCNs were taken with Zeiss Axio Imager Z2 microscope at 20× magnification. CSE: Cigarette smoke extract; BBR-LCN: Berberine-liquid crystalline nanoparticles.

The quantification of 5% CSE induced total ROS generation, and inhibition of ROS by BBR-LCNs in 16HBE cells were determined by DCF-DA fluorescence intensity and imaging. We observed that 5% CSE significantly increased the ROS production in 16HBE cells by >1.6-fold compared to CSE untreated group, while BBR-LCNs dose-dependently and significantly decreased the ROS generation (Figure 2A). Consistent with the fluorescence-intensity measurement, we also observed a similar trend in fluorescence imaging where BBR-LCNs significantly reduced the level of ROS intensity as observed by green fluorescence when compared to 5% CSE alone (Figure 2B).

### 3.4. Inhibition of CSE Induced Senescence of 16HBE Cells by BBR-LCNs

**Figure 3:**
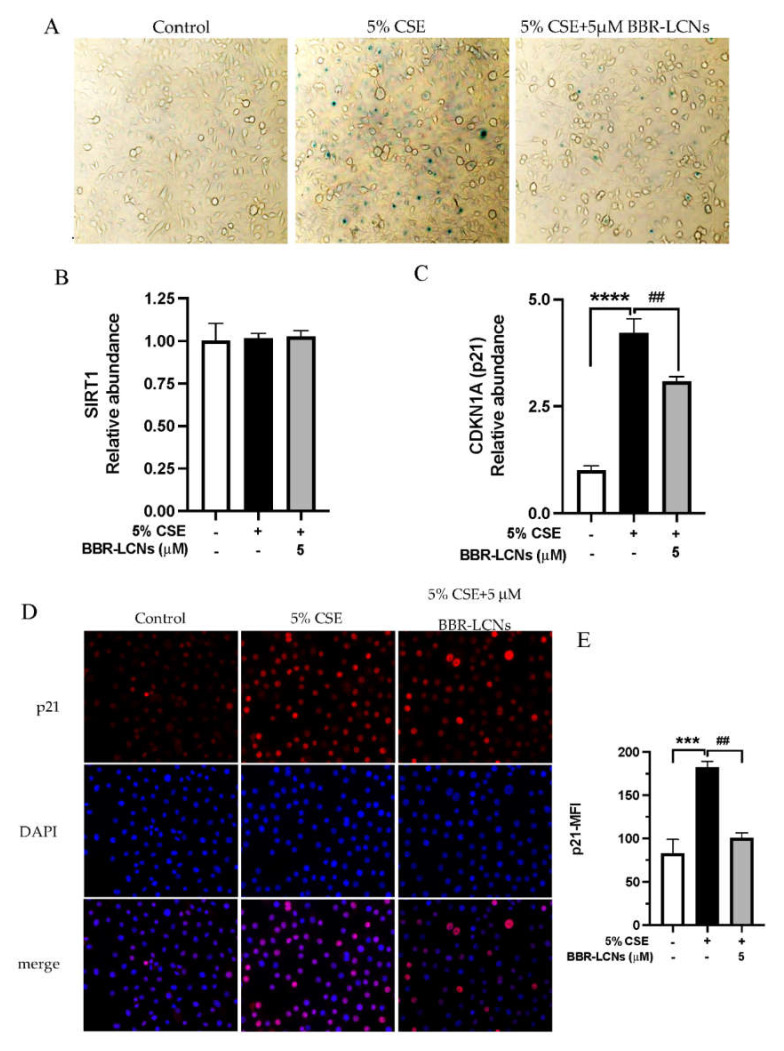
Effect of BBR-LCNs on 5% CSE-induced senescence of 16HBE. 16HBE cells treated with BBR-LCNs and 5% CSE for 24 h. (**A**) Cells were stained with b-galactosidase staining kit. Senescence-positive cells are represented with blue-colour positive staining of x-gal. Microscopic images were captured under a 20× magnification. Gene expression of (**B**) SIRT1 and (**C**) CDKN1A (p21). **** *p* < 0.0001 vs. control (without BBR-LCNs and 5% CSE treatment) and ^##^ *p* < 0.01 vs. 5% CSE. (**D**) Immunocytochemistry staining of p21-Alexa647; microscopic images were captured at 40× magnification. (**E**) Mean fluorescence intensity (MFI) of p21. *** *p* < 0.0001 vs control (without BBR-LCNs and 5% CSE treatment) and ^##^ *p* < 0.01 vs. 5% CSE.

Beta galactosidase staining was employed to determine cellular senescence induced by 5% CSE in 16HBE cells. As shown in Figure 3A, the microscopic image showed that a 24 h exposure of 5% CSE induced senescence of 16HBE, represented by senescence-positive, blue-stained cells. Treatment of BBR-LCNs for 24 h reduced the number of senescence-positive cells. For the mechanistic approach, we performed the RT-qPCR for gene expression of SIRT (antiaging) and p21 (senescence marker). Although there were no changes in SIRT expression in both 5% CSE and BBR-LCNs groups compared to untreated control (Figure 3B), the gene expression of *p21* was significantly upregulated by 5% CSE (4.2-fold) compared to untreated control. In contrast, BBR-LCNs significantly decreased the 5% CSE-induced p21 expression (Figure 3C). To further validate this with protein approach, we performed the immunocytochemistry staining of p21 and found that 5% CSE significantly induced p21 positive staining of 16HBE cells, while BBR-LCNs remarkably decreased the p21 fluorescence staining (Figure 3D).

### 3.5. Inhibition of Inflammation and Oxidative Stress-Related Gene Expression in 16HBE Cells by BBR-LCNs

**Figure 4:**
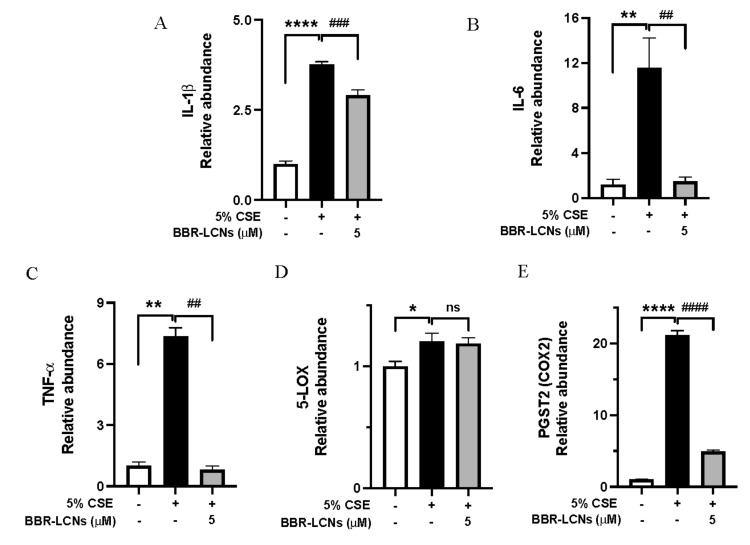

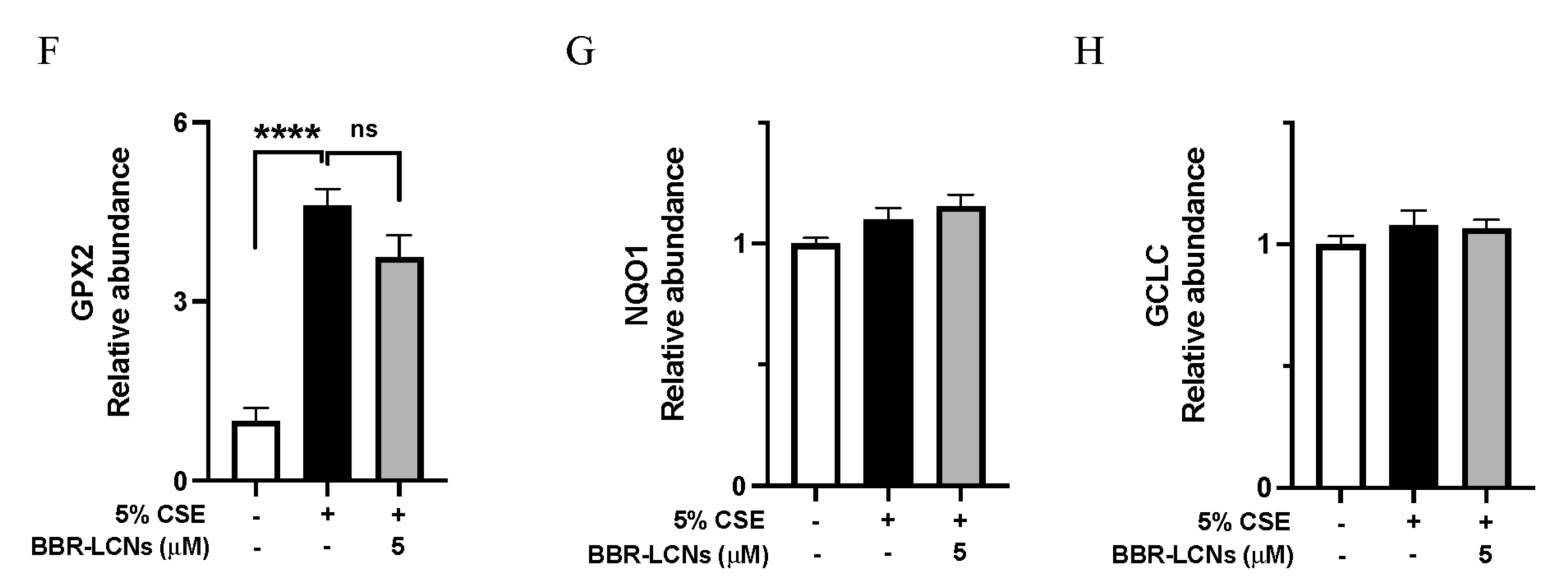
Expression of inflammation-related genes (**A**) *IL-1b*, (**B**) *IL-6*, (**C**) *TNF-a*, (**D**) *5-LOX*, (**E**) *PSGT2*, and oxidative-stress-related gene (**F**) *GPX2*, (**G**) *NQO1*, and (**H**) *GCLC* expression upon treatment with BBR-LCNs on 16HBE cells. Values are expressed as mean ± SEM (*n* = 4–6); * *p* < 0.05 ** *p* < 0.01, **** *p* < 0.0001 (control vs. 5%CSE) and ^##^ *p* < 0.01, ^###^ *p* < 0.001 ^####^ *p* < 0.0001 (5% CSE vs. 5% CSE + BBR-monoolein). ns = not significant. Analysis was performed by a one-way ANOVA followed by Tukey multiple comparison test.

**Figure 5:**
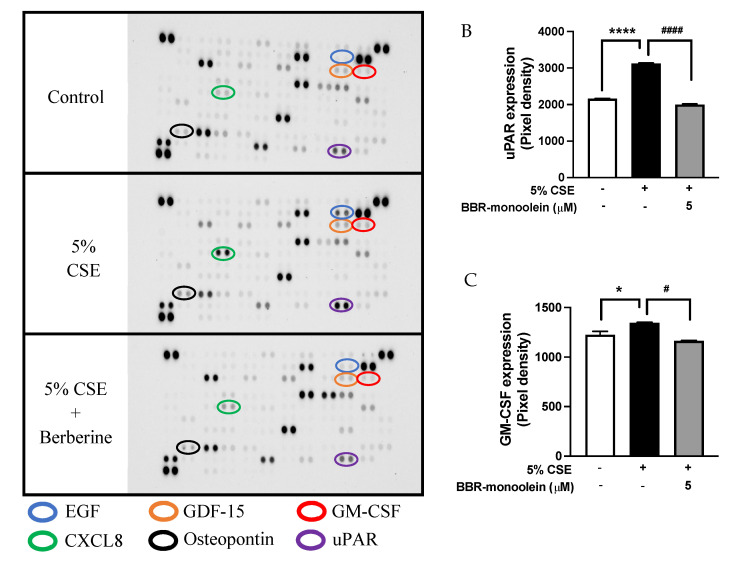

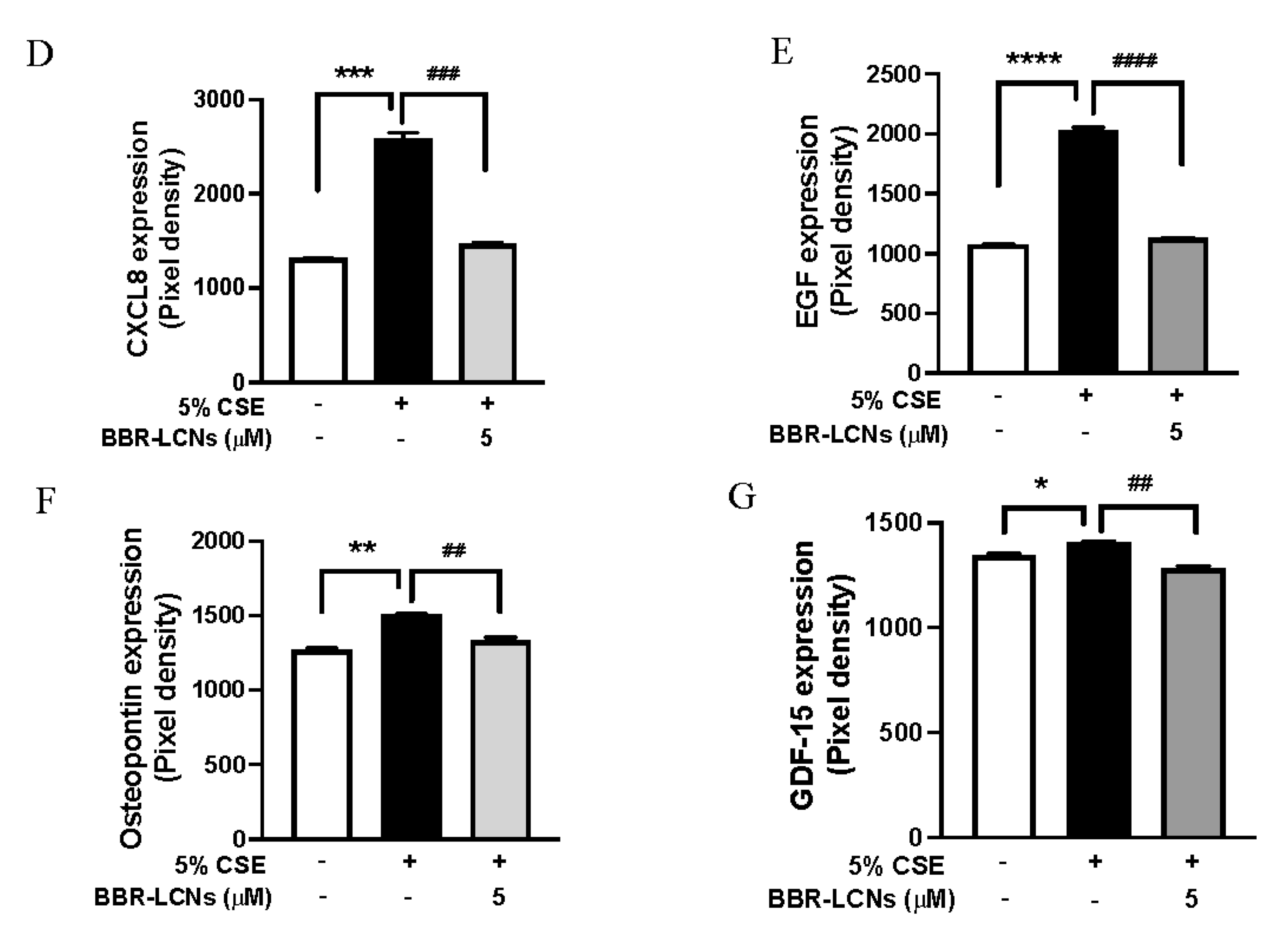
Expression of cytokines in human cytokine protein array. (**A**) protein blot, (**B**) uPAR, (**C**) GM-CSF (**D**) CXCL8, (**E**) EGF, (**F**) Osteopontin, and (**G**) GDF-15 upon treatment with BBR-LCNs on 16HBE cells. Values are expressed as mean ± SEM (n = 2–4); * *p* < 0.05, ** *p* < 0.01, *** *p* < 0.001, **** *p* < 0.0001 (control vs. 5% CSE) and ^#^ *p* < 0.05, ^##^ *p* < 0.01, ^###^ *p* < 0.001, ^####^ *p* < 0.0001 (5% CSE vs. 5% CSE + BBR-monoolein). Analysis was performed by a one-way ANOVA followed by Tukey multiple comparison test.

*IL-1β*, *IL-6*, and *TNF-α* are the main inflammatory cytokines overexpressed in cigarette smoking. In our results, 16HBE cells exposed to 5% CSE showed significant increase in *IL-1β* (3.77-fold)—Figure 4A, *IL-6* (11.5-fold)—Figure 4B, and *TNF-α* (7.3-fold)—Figure 4C compared to untreated control, while BBR-LCNs treatment at 5 μM notably decreased the gene expression compared with 5% CSE. Cyclooxygenase (COX)-2 and its product prostaglandin E2 are elevated in sputum of COPD patients, and they contribute to the severity of emphysema (airflow limitation) mediated by matrix metalloproteinase-2 during progression of COPD [39]. Similarly, 5-lipoxygenase (5-LOX) knockout mice exposed to cigarette smoke were protected from emphysema compared to air exposed, suggesting the inhibition of LOX-2 as a promising strategy to halt airway inflammation and oxidative stress [40]. While 5% CSE significantly increased the gene expression of both COX-2 (Figure 4E) and 5-LOX (Figure 4F) compared to CSE-untreated cells, the protective effect of BBR-LCNs was seen only by significant downregulation of COX-2 (but not 5-LOX).

Similarly, *Gpx2* is an antioxidant enzyme, and its expression during cigarette smoking will be higher to compensate the oxidative stress mediated by various oxidants in cigarette smoke. In our study, we observed that *Gpx2* expression was 4.6-fold higher in 5% CSE-treated 16HBE compared to untreated control, while BBR-LCNs decreased (but not significantly) the *Gpx2* expression (Figure 4F). The gene expression of NQO1 (Figure 4G) and GCLC (Figure 4H) was not significantly changed by both 5% CSE and BBR-LCNs.

### 3.6. Inhibition of 5% CSE Induced Cytokines Protein Expression in 16HBE Cells by BBR-LCNs

The protein expressions of uPAR, GM-CSF, CXCL8, EGF, Osteopontin, and GDF-15 are shown in Figure 5. As shown in the graph, 16HBE cells exposed to 5% CSE showed a significant increase in uPAR (1.44-fold)—Figure 5B, GM-CSF (1.1-fold)—Figure 5C, CXCL8 (1.95-fold)—Figure 5D, EGF (1.88-fold)—Figure 5E, Osteopontin (1.19-fold)—Figure 5F, and GDF-15 (1.05-fold)—Figure 5G compared to untreated control while BBR-LCNs treatment at 5 μM notably decreased the protein expression compared 5% CSE.

### 3.7. Inhibition of NO Production in RAW264.7 Cells by BBR-LCNs

**Figure 6:**
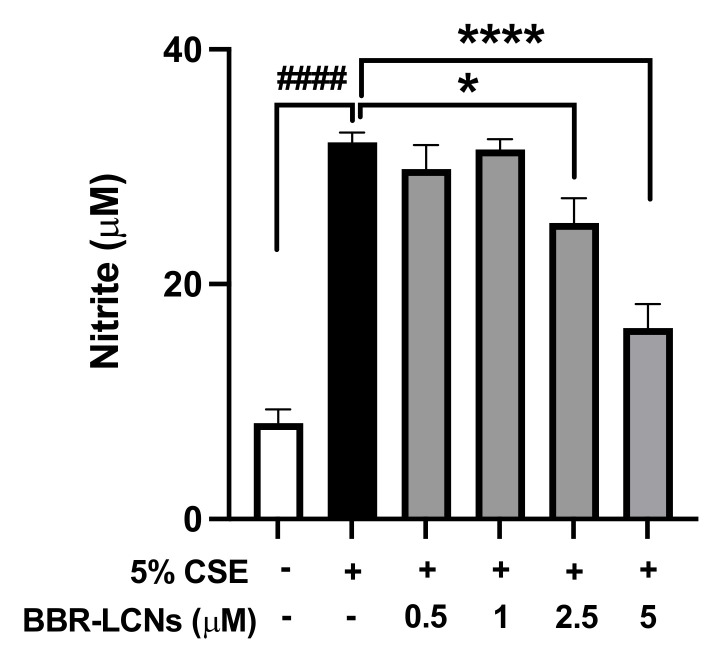
Effect of BBR-LCNs on NO production in RAW264.7 cells. NO production (in terms of nitrite) was quantified with Griess’ reagent by measuring absorbance at 540 nm. ^####^*p* < 0.0001 vs. control (without BBR-LCNs and 5% CSE treatment) and * *p* < 0.05; **** *p* < 0.0001 vs. 5% CSE. Values are expressed as mean ± SEM, n = 3 independent experiments. Analysis was performed with one-way ANOVA followed by Dunnett’s multiple comparison test.

We also measured the level of nitrite in the 5% CSE-induced RAW264.7 cells. As shown in Figure 6, there was a 4-fold increase in the level of nitrite by 5% CSE exposure for 24 h compared to the control (CSE untreated). The treatment of BBR-LCNs at doses of 2.5 and 5 μM significantly reduced the level of nitric oxide as compared to 5% CSE alone.

### 3.8. Inhibition of CSE-Induced ROS Generation in RAW264.7 Cells by BBR-LCNs

**Figure 7:**
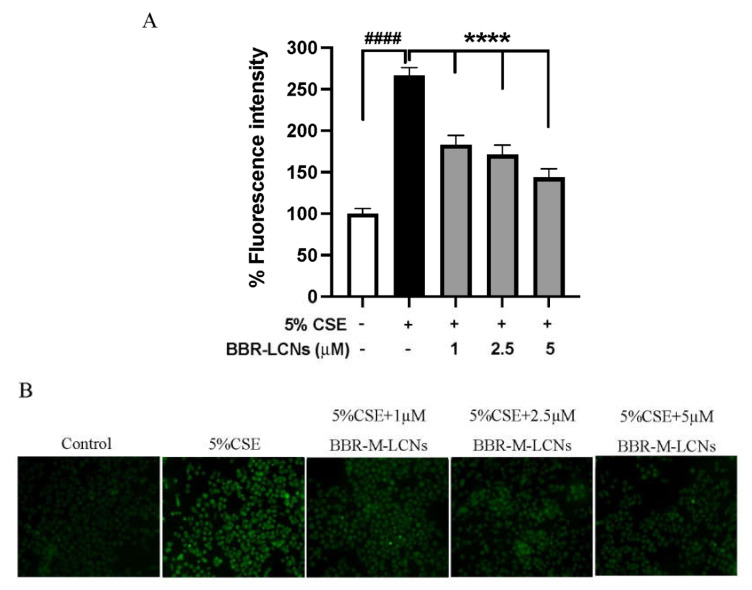
Effect of BBR-LCNs on ROS production in RAW264.7 cells. (**A**) Measurement of fluorescence intensity. The fluorescence intensity was measured at an excitation 485 nm and an emission 535 nm; n = 3 independent experiments and each independent experiment contained 6 replicates. ^####^ *p* < 0.0001 vs control (without BBR-LCNs and 5% CSE treatment) and **** *p* < 0.0001 vs. 5% CSE. Values are expressed as mean ± SEM. Analysis was carried out using one-way ANOVA followed by Dunnett’s multiple comparison test. (**B**) Fluorescence imaging. The DCF-DA fluorescence staining images of RAW264.7 cells treated with 5% CSE and with/without different concentrations of BBR-LCNs were captured with Zeiss Axio Imager Z2 microscope at 40× magnification.

The quantification of 5% CSE-induced total ROS generation and inhibition of ROS by BBR-LCNs in RAW264.7 cells were determined by DCF-DA fluorescence intensity and imaging. We observed that 5% CSE significantly increased the ROS production in RAW264.7 cells >2.6-fold compared to CSE-untreated group, while BBR-LCNs dose-dependently and significantly decreased the ROS generation (Figure 7A). Consistent with the fluorescence-intensity measurement, we also observed a similar trend in fluorescence imaging, where BBR-LCNs significantly reduced the level of ROS intensity as observed by green fluorescence when compared to 5% CSE alone (Figure 7B).

### 3.9. Inhibition of Oxidative Stress and Inflammation-Related Gene Expression in RAW264.7 Cells by BBR-LCNs

**Figure 8:**
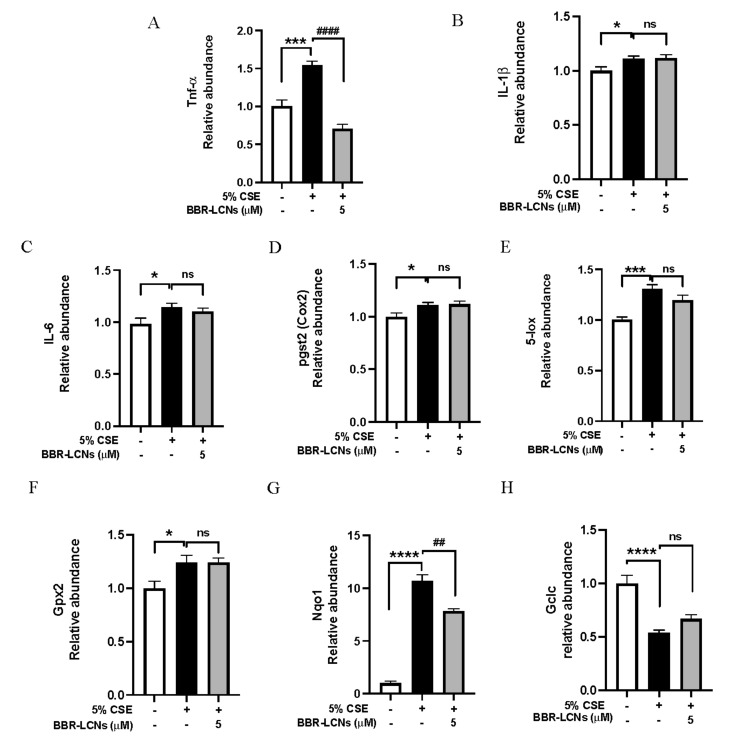
Inhibition of expression of inflammation-related gene (**A**) *Tnf-α*, (**B**) *IL-1β*, (**C**) *IL-6*, (**D**) *pgst2* (*cox2*), (**E**) *5-lox*, and oxidative-stress-related gene (**F**) *Gpx2*, (**G**) *Nqo1*, and (**H**) *Gclc* expression upon treatment with BBR-LCNs on RAW264.7 cells. Values are expressed as mean ± SEM (n = 4); * *p* < 0.05, *** *p* < 0.001, **** *p* < 0.0001 vs. control (without BBR-LCNs treatment) and ^##^ *p* < 0.01, ^####^ *p* < 0.0001 vs. 5% CSE only. ns = not significant. Analysis was performed by a one-way ANOVA followed by Tukey multicomparison test.

In our study, we observed that RAW264.7 cells exposed to 5% CSE showed significant increase in the expression of inflammatory gene *TNF-α* (Figure 8A), *IL-1β* (Figure 8B) and *IL-6* (Figure 8C), *cox-2* (Figure 8D), and *5-lox* (Figure 8E). In contrast, BBR-LCNs notably reduced the expression of *TNF-α* (but not *IL-1β*, *IL-6*, *cox-2*, and *5-lox*). Similarly, in case of oxidative-stress gene expression, 5% CSE decreases the *Gpx2* (Figure 8F), and *Gclc* (Figure 8G) expression and increases *Nqo1* expression compared to untreated control, while BBR-LCNs treatment at 5 μM showed no changes in *Gpx2* expression, an increase (but not significant) in the gene expression of *Gclc* (Figure 8H), and a significant decrease in *Nqo1* (Figure 8G) expression compared to 5% CSE only.

## 4. Discussion

**Figure 9:**
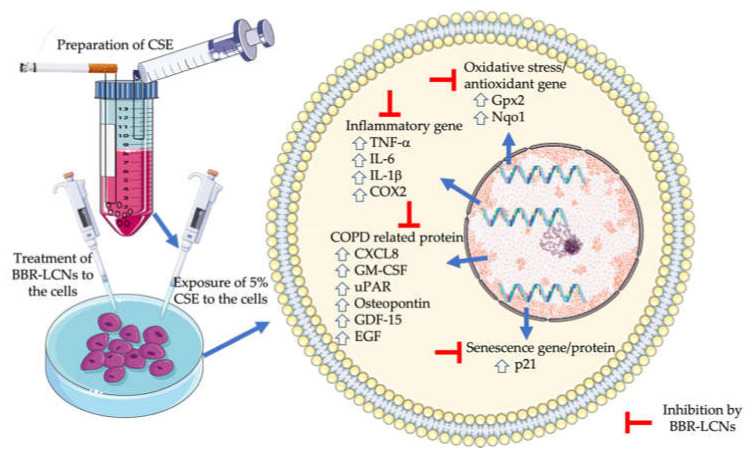
Antioxidative, anti-inflammatory, and antisenescence activity of BBR LCNs.

The significant observations in our study are the potent anti-inflammatory, antisenescence, and antioxidant activity of BBR-LCNs against cigarette-smoke-induced inflammation and oxidative stress in 16HBE and RAW264.7 and senescence in 16HBE cells. To reveal the mechanism behind this protective activity, we studied gene and protein expression associated with the changes after 5% CSE exposure (Figure 9).

In the quest for better therapeutic alternatives over synthetic drugs with various drawbacks such as off-target effects, complexity of route of administration, and affordability, researchers are now focusing on plant-based single purified biocompounds [41]. However, not every plant-based single moiety is an ideal therapeutic candidate. For example, the use of berberine itself as a therapeutic moiety is hindered by challenges such as poor oral bioavailability, low gastrointestinal absorption, and a high degree of elimination. Applying a nanotechnological approach, we have designed LCNs of berberine, and interestingly we have observed potent biological activity in vitro.

Scientific literature suggests that cigarette smoke is a strong inducer of airway inflammation, and the exposure of cigarette smoke to human broncho-epithelial cells and macrophages release various cytokines such as IL-1β, IL-6, and TNF-α [42]. In our study, the potent anti-inflammatory activity of BBR LCNs could be due to the inhibition of *IL-1β, IL-6,* and *TNF*-α gene expression in 16HBE cells and *TNF-a* gene expression, and NO production in RAW264.7 cells. The antioxidant role of BBR-LCNs in 16HBE was demonstrated by their ability to decrease the total ROS production. Generally, the generation of CSE-induced ROS are neutralised by a cellular defense system that involves the action of GCLC, GPX-2, HO-1, and NQO1. GCLC catalyses the formation of GSH, while GPX-2 catalyses the neutralisation of ROS by converting GSH to GSSG [43]. NQO1 and HO-1 are also responsible for protection against ROS and ROS-mediated oxidative damage [44,45]. However, scientific studies have also suggested that cells undergoing oxidative stress induced by CSE increase the expression of antioxidant genes as a cytoprotective response [46]. Bazzini et al. have shown that 24 h exposure of 5% CSE to 16HBE cells increases the *Gpx2* gene expression more than 3.5 fold (compared to CSE-untreated control) and this increase in *Gpx2* may be associated with a tolerance to cigarette smoking as a compensatory mechanism to combat cigarette-smoke oxidant [47]. In our study, we observed a 4.6-fold increase in *Gpx2* gene expression in 16HBE cells after 24 h exposure to 5% CSE compared to untreated control, while treatment of BBR-LCNs reduced (but not significantly) *Gpx2* expression compared to 5% CSE. In RAW264.7 cells, the target gene for antioxidant activity was *Nqo1*. The *Nqo1* gene was one among several smoking genes that overlapped between mice exposed to cigarette smoke and human smokers/COPD lung tissue. Gene expression was significantly upregulated in the lung tissue of smokers [48]. Pickett et al. observed a 5.73-fold increase in the expression of *Nqo1* gene in human bronchial epithelial cells after 18 h exposure of 5% CSE prepared from 2R4F (0.75 mg nicotine/cigarettes) reference cigarette [49]. Our 5% CSE prepared from 3R4F (0.75 mg nicotine/cigarettes) exposed to RAW264.7 cells for 24 h increased the *Nqo1* gene expression 10.6 fold. In contrast, treatment of BBR-LCNs significantly decreased the *Nqo1* expression. Apart from *Nqo1*, the antioxidant gene *Gclc* and *HO-1* expression was significantly decreased by 5% CSE and BBR-LCNs slightly increased (but not significantly) the expression. 

Furthermore, we also analysed the expression of COX-2 and 5-LO as a marker for pulmonary inflammation induced by 5% CSE. COX-2 is a rate-limiting enzyme in prostanoid pathway and has been associated with airway inflammation in COPD [39,50]. Likewise, 5-LO contributes to the production of proinflammatory leukotrienes via arachidonic acid metabolism and has been targeted for development of new therapies against COPD [51]. In our study, the mRNA levels of *COX-2* and *5-LO* was significantly upregulated by 5% CSE in 16HBE cells. However, only *COX-2* was downregulated by the BBR-LCNs in 16HBE cells (Figure 4E).

Targeting antiaging molecules such as SIRT [52] and senescence marker p21 and p16 expression could be a promising option for antisenescence activity of drugs [11]. In our study, the antisenescence activity of BBR-LCNs was primarily due to inhibition of both protein and gene expression of p21. The advantage of formulating berberine into LCNs offers great advantage over using free berberine powder for biological activity. Another researcher studying the anti-inflammatory activity of free berberine power in human airway epithelial cells observed significant activity at a dose of 25 μM (a 5-fold higher dose than our BBR-LCNs) [53]. Similarly, in the RAW264.7 cells, free or pure berberine powder at a dose ranging from 10–100 μM inhibited the expression of inflammation mediators such as IL-1β, IL-6, and TNF-α induced by LPS or LTB4 [54,55]. The potent biological activity of BBR-LCNs at a low dose compared to free berberine powder in published literature suggest that compounds such as berberine with unfavorable physiochemical characteristics can be improved by utilising a nanoformulation approach.

The limitations and future prospects of our study are outlined below. Firstly, as our experimental model is entirely in vitro, it would be interesting to explore the biological activity of berberine (via inhalation delivery) in an experimental COPD mice model induced by cigarette smoke. As our research group has developed a new short-term experimental model of COPD by exposing mice to cigarette smoke for 8–12 weeks [56,57], we are considering exploring this aspect in our upcoming future studies. Secondly, apart from lung epithelial cell and alveolar macrophage, there are many other cells in lungs such as fibroblasts, tracheal smooth-muscle cells, and goblet cells that could be explored. This would certainly enable an expansion to our investigations of cigarette-smoke-induced airway remodeling or fibrosis to be studied with the therapeutic potential of BBR-LCNs. Nevertheless, our study suggests that berberine can be a promising alternative for the attenuation of cigarette-smoke-induced airway inflammation, oxidative stress, and senescence of human bronchial epithelial cells, as well as halting the progression of airway inflammation to a chronic stage such as COPD. The potent biological activity of berberine and its formulation design have improved the physiochemical parameters and have enhanced its efficacy, stability, and cellular uptake.

## 5. Conclusions

The benefits of a nanotechnology-based approach to formulate free berberine into LCNs are clearly visible through its potent in vitro anti-inflammatory, antioxidant, and antisenescence activity against the 16HBE and RAW264.7 cell line. The anti-inflammatory activity of BBR-LCNs was due to the inhibition of *IL-1β, IL-6,* and *TNF*-α gene expression, while the antioxidant activity was due to the inhibition of total cellular ROS and associated genes (*Gpx2, Nqo1*). Similarly, the antisenescence activity of BBR-LCNs was due to the inhibition of p213 gene/protein expression. In conclusion, our study suggests BBR-LCNs as a promising alternative for the management of chronic lung disease. However, further detailed in vivo and clinical studies are essential to validate the findings.

## Figures and Tables

**Table 1:** List of primers used.

Cell Line	Gene Name	Forward Sequence	Reverse Sequence	Accession Number (RefSeqID)
16HBE	*SIRT1*	AAGGAAAACTACTTCGCAAC	GGAACCATGACACTGAATTATC	NM_001142498
	*CDKN1A(p21)*	CAGCATGACAGATTTCTACC	CAGGGTATGTACATGAGGAG	NM_000389
	*GPX2*	AATTTGGACATCAGCTGC	GGCTGCTCTTCAAGATTTAG	NM_000852
	*IL-1β*	GCCTCAAGGAAAAGAATCTG	GGATCTACACTCTCCAGG	NM_000576
	*IL-6*	GCAGAAAAAGGCAAAGAATC	CTACATTTGCCGAAGAGC	NM_000600
	*TNF-α*	AGGCAGTCAGATCATCTTC	TTATCTCTCAGCTCCACG	NM_000594
	*NQO-1*	AGTATCCACAATAGCTGACG	TTTGTGGGTCTGTAGAAATG	NM_000903
	*GCLC*	TTATTAGAGACCCACTGACAC	TTCTCAAAATGGTCAGACTC	NM_001197115
	*PTGS2 (COX-2)*	AAGCAGGCTAATACTGATAGG	TGTTGAAAAGTAGTTCTGGG	NM_000963
	*5-LOX*	AAATGCCACAAGGATTTACC	ATCGCTTTGGAGTAATTCAG	NM_000698
	*GAPDH*	TCGGAGTCAACGGATTTG	CAACAATATCCACTTTACCAGAG	NM_002046
RAW264.7	*Gclc*	CGACCAATGGAGGTGCAGTTA	AACCTTGGACAGCGGAATGA	NM_010295.2
	*Nqo1*	GTAGCGGCTCCATGTACTCTC	AGGATGCCACTCTGAATCGG	NM_008706.5
	*Tnf-α*	TCTGTCTACTGAACTTCGGGGTGA	TTGTCTTTGAGATCCATGCCGTT	NM_013693.3
	*IL-6*	AGAAAACAATCTGAAACTTCCAGAGAT	GAAGACCAGAGGAAATTTTCAATAGG	NM_031168
	*Gpx2*	ACC AGTTCGGACATCAGGAG	CCC AGGTCGGACATACTTGA	NM_030677
	*IL-1β*	TGGGATCCTCTCCAGCCAAGC	AGCCCTTCATCTTTTGGGGTCCG	NM_008361
	*Ptgs2* *(Cox-2)*	ACTCATAGGAGAGACTATCAAG	GAGTGTGTTGAATTCAGAGG	NM_011198
	*Alox5* *(5-Lox)*	CAGGAAGGGAACATTTTCATC	AGGAAGATTGGGTTACTCTC	NM_009662
	*Hprt*	AGGCCAGACTTTGTTGGATTTGAA	CAACTTGCGCTCATCTTAGGCTTT	NM_013556.2

## Data Availability

The data presented in this study are available on request from the corresponding author.
